# Current–Voltage Characteristics and Solvent Dissociation of Bipolar Membranes in Organic Solvents

**DOI:** 10.3390/membranes12121236

**Published:** 2022-12-07

**Authors:** Nobuyuki Onishi, Mie Minagawa, Akihiko Tanioka, Hidetoshi Matsumoto

**Affiliations:** 1 Department of Materials Science and Engineering, School of Materials and Chemical Technology, Tokyo Institute of Technology, 2-12-1 Ookayama, Meguro-ku, Tokyo 152-8552, Japan; 2 Interdisciplinary Cluster for Cutting Edge Research, Institute of Carbon Science and Technology, Shinshu University, 4-17-1, Wakasato, Nagano 380-8553, Japan

**Keywords:** bipolar membrane, organic solvent, current–voltage characteristic, solvent dissociation

## Abstract

In this work, the chronopotentiometric responses, pH changes, and current–voltage (*I*–*V*) characteristics of bipolar membrane (BPM)/LiCl–organic solvent systems were measured and compared with those of the BPM/LiCl–water system. Monohydric alcohols, polyhydric alcohols, and amides were used as organic solvents. The chronopotentiograms and pH changes supported that the organic solvents can dissociate into cations and anions at the BPM interface. It is found that amides cannot dissociate easily at the BPM compared with alcohols. The *I*–*V* characteristics showed that both the viscosity and acid–base property of organic solvents substantially influences the dissociation behaviors in addition to the autoprotolysis constant and relative permittivity of the solvents.

## 1. Introduction

Bipolar membranes (BPMs), which are composed of cation- and anion-exchange layers (CEL and AEL) joined together in series, show water-splitting behavior under a reverse bias condition. BPM electrodialysis (BPMED) is an efficient process for generating acids and bases without by-products and is applied to the recovery of acids and bases from wastewater or the production of organic and inorganic acids [1,2,3,4,5]. More recently, applications of BPMs into energy-related fields such as electrolysis cells and fuel cells have attracted much attention [4,5,6]. In addition, BPM is one of promising basic building blocks of “ionotronics” [5].

Solvent dissociation in BPMRD is not limited to water. Several research groups including our group reported alcohol dissociation into alkoxide anions and protons in BPMED as follows [7,8,9,10]:

MX + ROH → MOR + HX(1)

where ROH denotes an alcohol, and MOR and HX denote the corresponding alkali alkoxide and acid, respectively. This simple process can be used for green chemical syntheses, in particular, those using alkoxides to introduce alkoxyl groups to compounds (e.g., Claisen condensation and intramolecular Dieckman condensation) [11]. Our previous study indicated that the properties of monohydric and dihydric alcohols influence dissociation behaviors: solvents with a low autoprotolysis constant and high permittivity enhanced alcohol dissociation [8]. However, studies on the dissociation of organic solvent in BPMED have been quite limited and are still in the early stages [5]. The protonation–deprotonation mechanism of alcohols can be applied to other organic solvents (e.g., protic solvents). Therefore, the elucidation of the organic solvent dissociation mechanism will open a new direction in the applications of BPMED.

In this study, the current–voltage characteristics and the pH change during BPMED in organic solvent systems were evaluated. Herein, we extended the target organic solvents of BPMRD to trihydric alcohols and amides in addition to monohydric and dihydric alcohols. In particular, amides have unique properties different from alcohols: amides have a relative permittivity (*ε*_r_) larger than that of water and some of them have an autoprotolysis constant (p*K*_SH_) less than that of water (see Table 1). Eight kinds of organic solvents were used to investigate the influences of properties such as *ε*_r_, p*K*_SH_, and viscosity (*η*) on dissociation behaviors in BPMED.

## 2. Materials and Methods

### 2.1. Materials and Chemicals

A cation-exchange membrane (CEM, K501, Aciplex^®^) and anion-exchange membranes (AEM, A501, and A201, Aciplex^®^), which were supplied from Asahi Kasei Corporation [14], were used as the CEL and AEL, respectively. A superimposed-type BPM was prepared by placing K501 upon A501 in series as previously reported [15,16]. For amides, K501/A201 BPM was also prepared for comparison.

Methanol (MeOH), ethanol (EtOH), 1-propanol (PrOH), ethylene glycol (Et(OH)_2_), propylene glycol (Pr(OH)_2_), glycerine (Pr(OH)_3_), formamide (FA), and *N*-methylformamide (NMF) were used as solvents and lithium chloride (LiCl) was used as the electrolyte. All reagents (guaranteed reagent-grade or Wako special-grade) were purchased from Fujifilm Wako, Japan, and used without further purification. For reference, water (deionized water) was also used as a solvent. Deionized water was prepared using a water purifier (Pure-Line WL100, Yamato, Japan). The physicochemical properties of the solvents and IEMs are listed in Table 1 and Table 2, respectively.

### 2.2. Current–Voltage Characteristics and pH Change Measurements

Prior to the measurements, IEMs were immersed in 3 mol/L LiCl for 1 day to ensure that the counterions were exchanged with Li^+^. After sufficiently washing the membranes in deionized water, the IEMs are immersed in a pure organic solvent for 1 week. Thereafter, the IEMs were equilibrated with 0.1 mol/L LiCl solutions.

**Figure 1:**
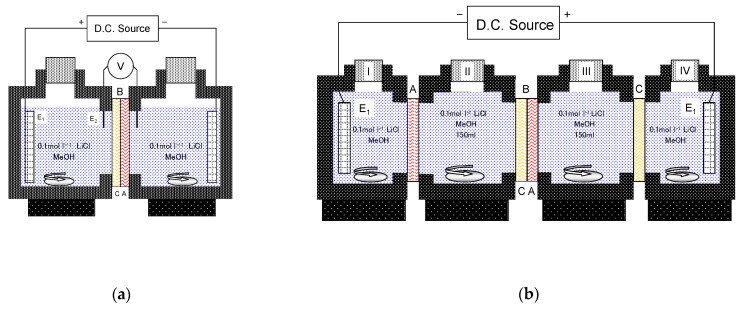
(**a**) Schematic diagram of typical apparatus for the current–voltage measurements. B is the BPM (C and A are CEL and AEL, respectively), E_1_ is Pt electrode, and E_2_ is Ag/AgCl electrode. The current–voltage characteristics in the other organic solvent systems were measured in the same manner. (**b**) Schematic diagram of typical apparatus for measuring the pH shift. B is the BPM (C and A are CEL and AEL, respectively), C is CEM, A is AEM, and E_1_ is Pt electrode. The current–voltage characteristics in the other organic solvent systems were measured in the same manner.

The chronopotentiometric responses and current–voltage characteristics of the BPMs were investigated by controlled-current voltammetry using a two-compartment glass cell (Figure 1a) [8]. The BPM (Aciplex K501/A501 or K501/A201, effective area of 3.14 cm^2^) was placed between the two compartments, which were both filled with 0.1 mol/L LiCl solutions. Both compartments were well-stirred using magnetic stirrers. The potential drop (*V*_measure_) across the BPM was measured by Ag/AgCl wire electrodes (diameter of 0.1 mm) placed on both sides of the membrane connected to a voltmeter (Fluke 45 Multimeter, Everett, WA, USA) under conditions where a current was supplied by Pt-black disc electrodes connected to a DC current source/monitor (Advantest TR6143, Chiyoda-ku, Tokyo, Japan). All measurements were carried out at 25 ± 1 °C. Under a reverse bias condition, both the current and voltage are negative and the curve is displayed in the third quadrant.

For the pH measurements, a four-compartment glass cell (Figure 1b) was used [8]. The BPM (K501/A501, effective area of 3.14 cm^2^), AEM (A501, effective area of 7.07 cm^2^), and CEM (K501, effective area of 7.07 cm^2^) were placed between the two compartments, II and III, I and II, and III and IV, respectively. Compartments II and III were both filled with 150 mL of 0.1 mol/L LiCl solutions; and compartments I and IV were both filled with 24 mL of 0.1 mol/L LiCl solutions. In general, the smaller-volume compartments are required to reduce the resistance of the solution during ED. Here, the compartments II and III with a larger volume were used to obtain a sufficient volume of samples for the pH measurements. All compartments were well-stirred using magnetic stirrers. The current was supplied by Pt-black disc electrodes connected to a DC current source/monitor (Advantest TR6243, Chiyoda-ku, Tokyo, Japan). All measurements were carried out at 25 ± 1 °C. After applying a constant current for 1 hr, the pH of the solutions collected from compartments II and III was measured by a pH meter (TOA WM-50EG, Shinjuku-ku, Tokyo, Japan) equipped with a combination pH electrode (TOA GST-5721S, for organic solvent, Shinjuku-ku, Tokyo, Japan) under a nitrogen atmosphere. The combination pH electrode contains an internal reference electrode in ionic contact with a glass electrode (GE) as the potentiometric pH sensor, and an external reference electrode (RE, a Ag/AgCl electrode), where GE is immersed in the solution under the test and RE immersed in 3.3 mol/L KCl aqueous solution, in a single probe. The acid–base property in nonaqueous solvents can be measured using the same definition as aqueous solutions: pH = −log*a*_H_ (*a*_H_ is the activity of protons in a given solvent) [17,18]. Each nonaqueous solvent has its own solvent-specific pH scale, ^s^pH (the superscript s denotes the solvent). The pH window is fixed by the autoprotolysis constant (*K*_SH_) of a given solvent. Note that the instrumental pH value obtained from the pH meter calibrated by aqueous standard solutions should be corrected by considering the diffusion potential between aqueous filling of the RE and outer organic solution and the energy of proton transfer from an infinitely diluted aqueous solution to the same nonaqueous solution [17]. Therefore, it is impossible to directly compare the acid–base properties between different solvents from the instrumental pH values obtained from the pH meter. Here, we used the change in the instrumental pH value during ED (ΔpH = pH_after1hr-ED_ − pH_initial_) to evaluate changes in the solution state: a positive ΔpH value indicates the basic shift of the solution and vice versa.

## 3. Results and Discussion

### 3.1. Chronopotentiograms and pH Changes in BPM/Organic Solvent Systems

**Figure 2:**
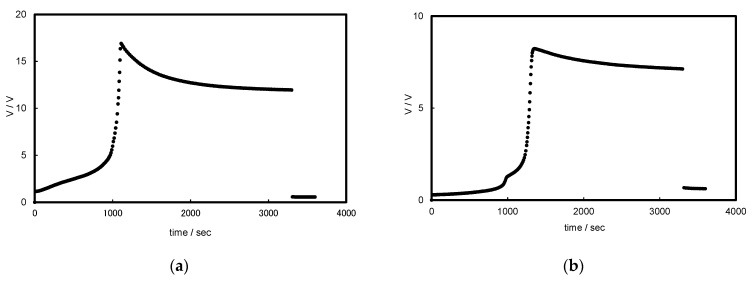

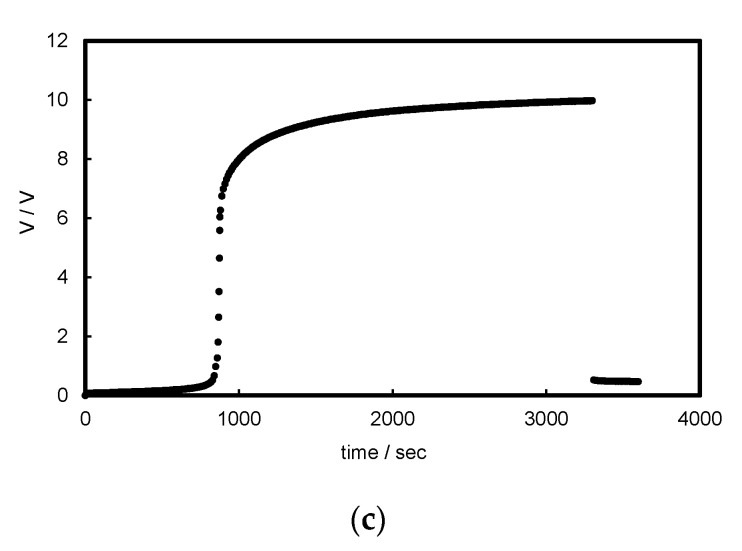
Typical chronopotentiometric responses for (**a**) BPM/EtOH, (**b**) BPM/Et(OH)_2_, and (**c**) BPM/FA systems at the constant current density of 0.16 mA cm^−1^.

At first, typical chronopotentiograms in BPM/organic solvent systems (i.e., BPM/EtOH, BPM/Et(OH)_2_, and BPM/FA) are shown in Figure 2. For alcohols (Figure 2a,b), the chronopotentiograms showed similar responses to the BPM/water and BPM/MeOH systems at a high current density (Figure S1c,d, Supplementary Material). The voltage drastically increased due to the depletion of co-ions at the BPM interface. Then, solvent dissociation occurred and the generated protons and alkoxide anions carried charges, resulting in decreases in the BPM resistance and voltage. For amides (Figure 2c), on the other hand, the chronopotentiograms showed similar responses to the BPM/water and BPM/MeOH systems at a low current density (Figure S1a,d, Supplementary Material). The voltage at the BPM interface is not high enough to dissociate the solvents effectively. In addition to the solvent dissociation, transport of co-ions (Li^+^ and Cl^−^) through the respective CEL and AEL occurred, causing a gradual increase in the voltage.

Next, to confirm the dissociation of organic solvents at the BPM interface in a different way, the pH change in the solution phases was measured after BPMED. The dissociation reaction for EtOH, Et(OH)_2_, FA, and NMF as the typical organic solvent systems are expressed as follows:

(2) $$2C_{2}H_{5}\mathrm{OH}↔C_{2}H_{5}{\mathrm{OH}}_{2}^{+}+C_{2}H_{5}O^{-}$$

(3) $$2{\mathrm{HOC}}_{2}H_{4}\mathrm{OH}↔{\mathrm{HOC}}_{2}H_{4}{\mathrm{OH}}_{2}^{+}+{\mathrm{HOC}}_{2}H_{4}O^{-}$$

(4) $$2{\mathrm{HCONH}}_{2}↔{\mathrm{HCONH}}_{3}^{+}+{\mathrm{HCONH}}^{-}$$

(5) $$2{\mathrm{HCONHCH}}_{3}↔{\mathrm{HCONH}}_{2}^{+}\left({\mathrm{CH}}_{3}\right)+{\mathrm{HCONH}}^{-}\left({\mathrm{CH}}_{3}\right)$$

Thus, the cations and anions were generated at the BPM interface and permeated through the CEL and AEL into the solution phases, respectively.

**Figure 3:**
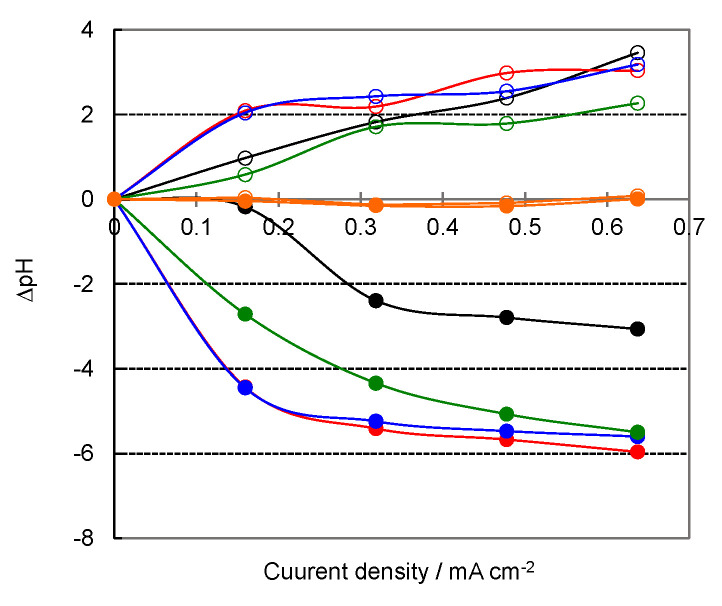
The pH change (ΔpH) in the solution phases on the CEL (solid circles) and AEL (open circles) sides after the 1 h BPMED as a function of current density: H_2_O (●), MeOH (●), EtOH (●), Et(OH)_2_ (●), and FA (●).

Figure 3 shows the pH changes (ΔpH) in the solution phases on the CEL and AEL sides after the 1 hr BPMED at various current densities. The ΔpH values for water and alcohol systems increased on the AEL side but decreased on the CEL side as the current density increased, indicating that more solvent molecules dissociated at the BPM interface and the protons and anions permeated through the CEL and AEL, respectively. The reason for the lower ΔpH values on the AEL side compared to those on the CEL side is due to the dissolution of atmospheric CO_2_ into the solution. For the FA system, on the other hand, almost no pH change was observed in both the solution phases on the CEL and AEL sides. Then, we evaluated the pH change after applying the current to the cell for 8 hrs at a density of 0.64 mA cm^−2^. The pH changes for amide systems were smaller than those in the aqueous and alcohol systems (Table 3), suggesting that there were fewer dissociation products. This would be due to the properties of amides. We discuss the reason in the later section.

The chronopotentiometry and pH changes supported that the organic solvent can dissociate at the BPM interface. We think that dissociation reactions such as those in Equations (2)*–*(5) occurred at the BPM interface. For the BPM/MeOH and BPM/EtOH systems, the production of methoxide and ethoxide was experimentally confirmed [8,9]. Here, we could not carry out direct characterization of the products due to the limitation of generated products.

### 3.2. Current–Voltage Characteristics in BPM/Organic Solvent Systems

**Figure 4:**
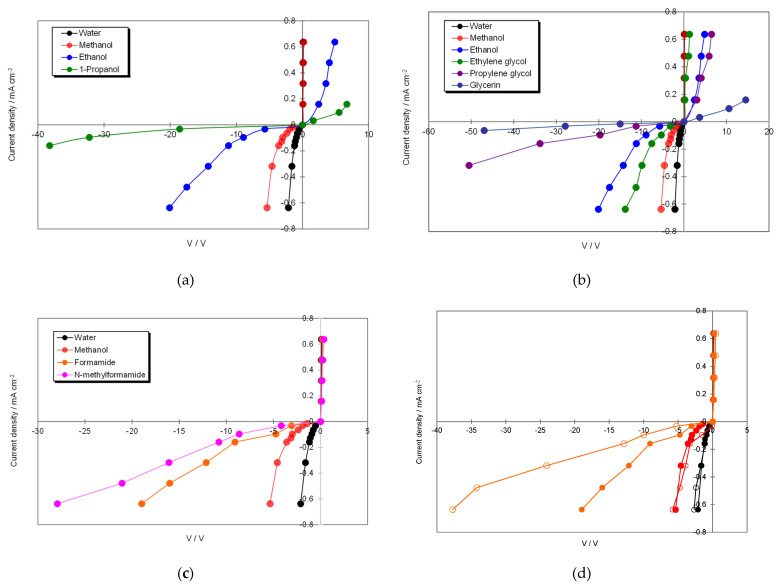
The current density (*J*)–voltage (*V*) curves of the BPMs in (**a**) monohydric alcohols, (**b**) dihydric and trihydric alcohols, and (**c**) amides. (**d**) *J*–*V* curves of the K501/A501 BPM (solid circles) and K501/A201 BPM (open circles) in H_2_O (●), MeOH (●), and FA (●).

Figure 4 shows the current density–voltage (*J*–*V*) curves of the BPMs in alcohols and amides. All curves demonstrated the rectification and solvent dissociation behaviors: for the forward voltages (V > 0), the current was carried by the salt ions and increased with the voltage, while for relatively small reverse voltages (V < 0), the current was also carried mainly by the salt ions and attained a limiting value. The resistances of the BPMs in EtOH, PrOH, Pr(OH)_2_, and Pr(OH)_3_ were very large. At high reverse voltages, most of the current was carried by the cations and anions generated by solvent dissociation (e.g., Equations (2)–(5)) at the BPM interface and increased rapidly with the voltage.

For monohydric alcohols (Figure 4a), the order of the voltage required for solvent dissociation was H_2_O < MeOH < EtOH < PrOH. This trend agreed well with the order of pK_SH_ (H_2_O < MeOH < EtOH < PrOH). Detailed discussion on the influence of ε_r_ on solvent dissociation is described later.

For dihydric and trihydric alcohols (Figure 4b), the order of the voltage required for solvent dissociation, which was Pr(OH)_3_ > Pr(OH)_2_ >> Et(OH)_2_ > EtOH > MeOH, did not agree with those of ε_r_ and pK_SH_ of the solvent (Table 1). We, however, can explain the order of the voltage required for solvent dissociation using that of viscosity: Pr(OH)_3_ (1412 mPas) > Pr(OH)_2_ (56 mPas) >> Et(OH)_2_ (20 mPas) > EtOH (1.2 mPas) > MeOH (0.6 mPas) (Table 1). These findings clearly indicate that the viscosity of the solvents substantially contributes to the solvent transport through the CEL and AEL and, consequently, the dissociation behaviors at the BPM interface.

For amides (Figure 4c), the voltage required for solvent dissociation is much higher than that in water and MeOH, indicating that the dissociation efficiency of amides was quite low. These results were not contradictory to those from the chronopotentiograms and pH changes. In addition, the amide dissociation behaviors depended on the material of AEL (see Figure 4d, the difference between K501/A501 and K501/A201 was substantial only for amides). This would be due to the strong basicity of amides. According to Kolthoff’s classification [19], MeOH and Et(OH)_2_ are in neutral amphiprotic solvents with moderate acidity and basicity similar to water, whereas FA and NMF are in protophilic amphiprotic ones with weak acidity and strong basicity. In general, it is known that strong bases in aqueous solutions exhibit weakly basic behavior in strongly basic solvents. Therefore, the actual effectiveness of the fixed-charge groups in the membrane (in particular, in AEL) is significantly weaker in amides than in aqueous and alcohol solutions. In addition, protophilic amphiprotic solvents do not tend to release protons due to the stronger basicity than that of neutral amphiprotic ones. Thus, proton dissociation between the fixed-charged groups and the solvent molecules would be difficult to achieve.

Simons suggested that the hydronium and hydroxyl ions are produced from the protonation–deprotonation between some functional groups and water molecules and proposed the following mechanism for the water dissociation reaction [20,21,22]:

Under a high electric field (*E*) at the BPM interface, the dissociation rate constant (*k*_d_) in the proton exchange reaction at the BPM interface can be written as:

(6) $$k_{d}=k_{d}^{0}\exp\left(\frac{\alpha FE}{RT}\right)$$

where $k_{d}^{0}$ is the dissociation rate constant at the BPM interface without an electric field, α is the distance factor, *F* is the Faraday constant, *E* is the electric field intensity at the BPM interface, *R* is the gas constant, and *T* is the absolute temperature. $k_{d}^{0}$ can be expressed using the Arrhenius equation:

(7) $$k_{d}^{0}=B\exp\left(-\frac{E_{a}}{RT}\right)$$

where *B* is the frequency factor, *E*_a_ is the activation energy.

*E* can be also written as follows [2,23]:

(8) $$E=\sqrt{\frac{2F}{\varepsilon_{0}\varepsilon_{r,\mathrm{interface}}}}\left(-V_{\mathrm{interface}}\frac{X_{\mathrm{CEL}}X_{\mathrm{AEL}}}{X_{\mathrm{CEL}}+X_{\mathrm{AEL}}}\right)$$

where *ε*_0_ and *ε*_r,interface_ are the vacuum permittivity and relative permittivity at the BPM interface, respectively, *V*_interface_ is the potential difference at the BPM interface (*V*_interface_ = *V*_measure_ − (*V*_CEL_ + *V*_AEL_) = *V*_measure_ − *i*(*R*_CEL_ + *R*_AEL_), *R* denotes resistance), and *X*_CEL_ and *X*_AEL_ are charge densities in CEL and AEL, respectively. Our theoretical predictions clearly show that the normalized dissociation rate ($k_{d}/k_{d}^{0}$) increases with a decrease in *ε*_r,interface_ (Figure S2, Supplementary Material). For simplification, we used the *K*_SH_ of the organic solvents as $k_{d}^{0}$.

Water molecules undergo autoprotolysis (self-ionization) as follows:

(9) $$2H_{2}O↔H_{3}O^{+}+{\mathrm{OH}}^{-}$$

(10) $$K_{w}=a\left(H_{3}O^{+}\right)a\left({\mathrm{OH}}^{-}\right)$$

where *K*_w_ is the product of activities and its negative 10 logarithm is expressed as p*K*_SH_. Similarly, the amphiprotic solvent molecules (SH) such as MeOH, EtOH, and FA undergo autoprotolysis as well as water molecules. In general, *K*_SH_ is defined as follows:

(11) $$2\mathrm{SH}↔{{\mathrm{SH}}_{2}}^{+}+S^{-}$$

(12) $$K_{\mathrm{SH}}=a\left({{\mathrm{SH}}_{2}}^{+}\right)a\left(S^{-}\right)$$

p*K*_SH_ indicates the ease of dissociation of the solvent and a small p*K*_SH_ value tends to give a large $k_{d}^{0}$ one. The p*K*_SH_ values of the solvents used here are listed in Table 1.

As explained above, for the monohydric alcohols, the order of the ease of solvent dissociation was H_2_O < MeOH < EtOH < PrOH. This trend agreed well with the order of p*K*_SH_ (H_2_O < MeOH < EtOH < PrOH), but contradicts the theoretical prediction (Figure S2, Supplementary Material) based on the order of *ε*_r_ (H_2_O > MeOH > EtOH > PrOH). It is difficult to experimentally determine *ε*_r,interface_ in actual BPM/organic solvent systems. Thus, in order to more precisely consider the relative permittivity at the BPM interface (*ε*_r,interface_) in Equation (8), we adopted the relative permittivity of the solvent in the membranes (*ε*_r, local_) instead of that in the bulk solvent (*ε*_r_). Bruggeman presented the local permittivity (i.e., *ε*_r,local_) in the membranes as follows [24]:

(13) $$\frac{\varepsilon_{r,\mathrm{local}}-\varepsilon_{r}}{\varepsilon_{m}-\varepsilon_{r}}\sqrt[{3}]{\frac{\varepsilon_{m}}{\varepsilon_{r,\mathrm{local}}}}=1-\phi$$

where *ε*_r,local_, *ε*_r_, and *ε*_m_ are relative permittivity of the solvent in the membranes, the relative permittivity of the solvent, and the relative permittivity of the membrane matrix (=5), respectively, and ϕ is the volume fraction of the solvent in the membrane. The calculated *ε*_r,local_ values for the volume fraction ϕ of 0.5 using Equation (13) were 13, 12, and 10 for MeOH, EtOH, and PrOH, respectively (Table S1, Supplementary Material). For the alcohol systems, the differences in the *ε*_r,local_ values between the solvent and water systems were small, indicating that the contribution of *ε*_r,local_ is negligible and that of p*K*_SH_ (i.e., $k_{d}^{0}$) is more substantial. On the contrary, for the amide systems, the *ε*_r,local_ values of amides (FA of 23 and NMF of 27 for ϕ of 0.5) were higher than that of water (20 for ϕ of 0.5). A large *ε*_r,local_ value would cause an increase in the voltage required for solvent dissociation based on the theoretical prediction via a decrease in *k*_d_ (Figure S2, Supplementary Material, we regard *ε*_r,local_ as *ε*_r,interface_.).

## 4. Conclusions

In this work, organic solvent dissociation behaviors were investigated by BPMED in organic solvent systems (i.e., monohydric alcohols, polyhydric alcohols, and amides). Alcohols can dissociate at the BPM interface, while amides cannot dissociate easily. Our analyses clearly indicate that both the viscosity and acid–base property of organic solvents substantially influence the dissociation behavior in addition to p*K*_SH_ and *ε*_r_ of the organic solvents. For monohydric alcohols, the effect of p*K*_SH_ was significant, but that of *ε*_r_ was not substantial. For polyhydric alcohols, on the other hand, the effect of the viscosity is more significant compared with that of p*K*_SH_. The dissociation efficiency of amides was quite low and this would be due to the strong basicity of amides. These results provide fundamental information for the understanding of dissociation behaviors of organic solvents in BPMED. We believe that these findings and the development of robust BPMs with a low organic solvent resistance create a new field of applications for BPMs in the future.

## Figures and Tables

**Table 1:** Physicochemical properties of the solvents [12,13].

Solvent	Chemical Formula	*ε*_r_*^a^* [-]	p*K*_SH_ *^b^* [-]	Viscosity [mPas]
Water	H_2_O	78.4	14.00	1.00
Methanol (MeOH)	CH_3_OH	32.7	16.71	0.611
Ethanol (EtOH)	CH_3_CH_2_OH	24.6	18.90	1.19
1-propanol (PrOH)	CH_3_CH_2_CH_2_OH	20.5	19.43	2.20
ethylene glycol (Et(OH)_2_)	HOCH_2_CH_2_OH	37.7	15.84	19.9
propylene glycol (Pr(OH)_2_)	CH_3_CH(OH)CH_2_OH	32.0	17.21	56.0
Glycerin (Pr(OH)_3_)	HOCH_2_CH(OH)CH_2_OH	42.5	-	1412
Formamide (FA)	HCONH_2_	111.0	16.80	3.75
*N*-methylformamide (NMF)	HCONHCH_3_	182.4	10.74	-

*^a^ ε*_r_: relative permittivity. *^b^ K*_SH_: autoprotolysis constant.

**Table 2:** Physicochemical properties of ion-exchange membranes (IEMs) used for BPMs.

Membrane	Matrix *^a^*	Reinforcement *^b^*	Ion-Exchange Groups	Ion-Exchange Capacity [mmol/g-dry membr.]
K501 (CEM)	poly(St-*co*-DVB)	PVC woven fabric	sulfonic acid	2.0
A501 (AEM)	poly(St-*co*-DVB-*co*-CMS)	PVC woven fabric	quaternary alkyl ammonium	1.8
A201 (AEM)	Poly(St + DVB + VI)	PP woven fabric	quaternary imidazole	1.4

*^a^* St: styrene; DVB: divinylbenzene; CMS: chloromethylstyrene; VI: vinylimidazole. *^b^* PVC: poly(vinylchloride); PP: poly(propylene).

**Table 3:** The pH change (ΔpH) for amide systems.

Solvent	ΔpH_CEL_	ΔpH_AEL_
FA	−0.21	0.46
NMF	−0.56	0.07

## Supplementary Materials

The following supporting information can be downloaded at: https://www.mdpi.com/article/10.3390/membranes12121236/s1, Figure S1: Typical chronopotentiometric responses for the BPM/water system at the constant current densities of (a) 0.01, (b) 0.16, and (c) 0.64 mA cm^–2^ and (d) for the BPM/MeOH system at the constant current densities ranging from 0.06 to 0.64 mA cm^–2^; Figure S2: Effect of dielectric constant *ε*_r,interface_ at the BPM interface on the normalized dissociation rate constant ($k_{d}/k_{d}^{0}$) calculated using the chemical reaction model; Table S1: The local dielectric constants *ε*_r,local_ in the membrane calculated by the Bruggeman’s equation.
