# Supplementary material for: Current–Voltage Characteristics and Solvent Dissociation of Bipolar Membranes in Organic Solvents
Source: Membranes (Basel). 2022 Dec 7;12(12):1236. doi: 10.3390/membranes12121236 (PMC9781749; doi:10.3390/membranes12121236)
Supplement: Supplementary file 1 [file membranes-12-01236-s001.zip › membranes-2063159-supplementary.pdf]

Supplementary Material: Current-Voltage Characteristics and Solvent Dissociation of Bipolar Membranes in Organic Solvents

Nobuyuki Onishi, Mie Minagawa, Akihiko Tanioka and Hidetoshi Matsumoto*

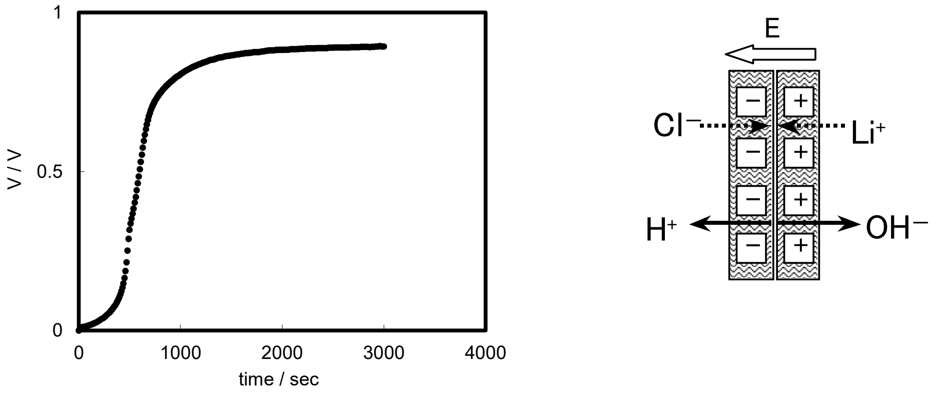

(**a**)

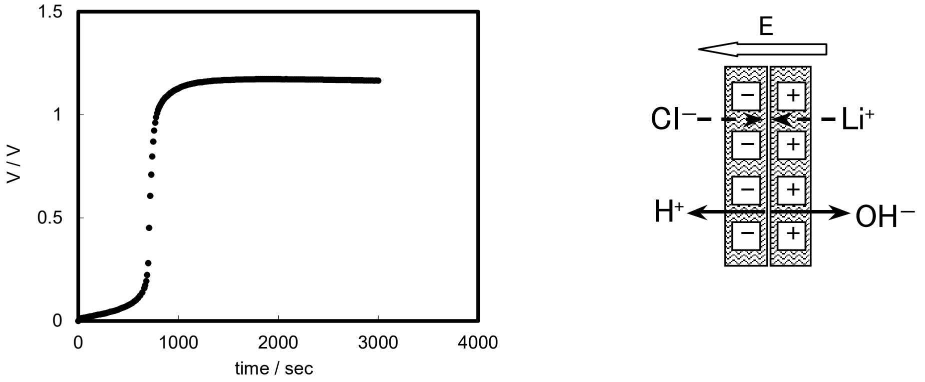

(**b**)

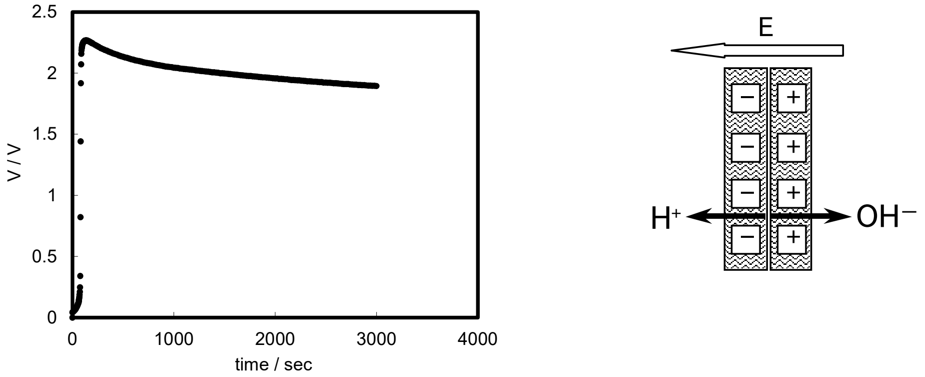

(**c**)

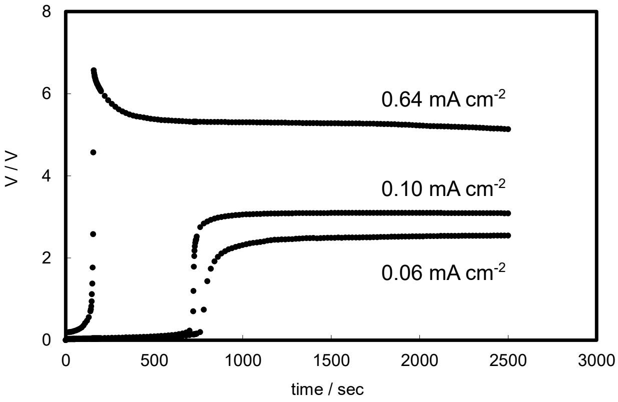

(d)

Figure S1. Typical chronopotentiometric responses for the BPM/water system at the constant current densities of (a) 0.01, (b) 0.16, and (c) 0.64 mA cm^{-2} and (d) for the BPM/MeOH system at the constant current densities ranging from 0.06 to 0.64 mA cm^{-2}.

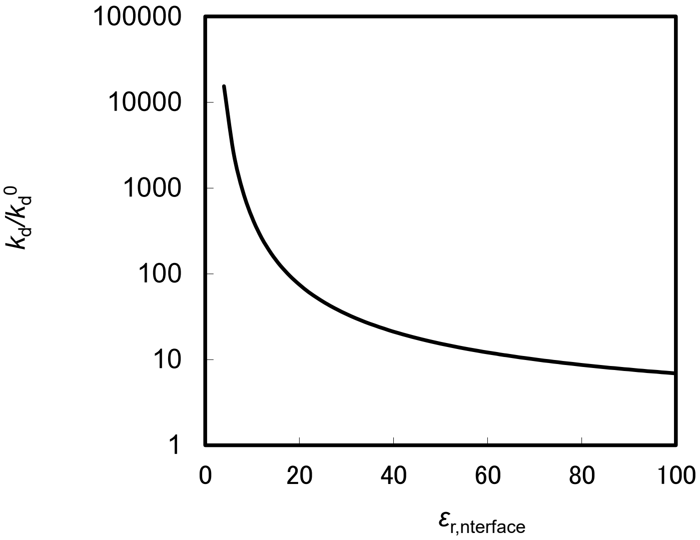

Figure S2. Effect of dielectric constant $\varepsilon_{r,\mathrm{interface}}$ at the BPM interface on the normalized dissociation rate constant (k_d/k_d^0) calculated using the chemical reaction model.

Table S1. The local dielectric constants $\varepsilon_{r,local}$ in the membrane calculated by the Bruggeman's equation.

Solvent	In solution (ϕ = 1)	In membrane (ϕ = 0.5)
Water	78.4	20
Methanol (MeOH)	32.7	13
Ethanol (EtOH)	24.6	12
1-propanol (PrOH)	20.5	10
ethylene glycol ($Et(OH)_2$)	37.7	14
propylene glycol ($Pr(OH)_2$)	32.0	13
Glycerin ($Pr(OH)_3$)	42.5	15
Formamide (FA)	111.0	23
N-methylformamide (NMF)	182.4	27
